# The role of whole-body computed tomography in the diagnosis of thoracic injuries in severely injured patients – a retrospective multi-centre study based on the trauma registry of the German trauma society (TraumaRegister DGU^®^)

**DOI:** 10.1186/s13049-017-0427-4

**Published:** 2017-08-15

**Authors:** Patricia Lang, Martin Kulla, Fabian Kerwagen, Rolf Lefering, Benedikt Friemert, Hans-Georg Palm, TraumaRegister DGU

**Affiliations:** 1 Trauma Research Group, Department of Orthopaedics and Trauma Surgery, Reconstructive and Septic Surgery, and Sports Traumatology, German Armed Forces Hospital of Ulm, Ulm, Germany; 2 Department of Anaesthesiology and Intensive Care Medicine, German Armed Forces Hospital of Ulm, Oberer Eselsberg 40, 89081 Ulm, Germany; 3 Institute for Research in Operative Medicine (IFOM), Witten/Herdecke University, Ostmerheimer Str. 200, 51109 Cologne, Germany

**Keywords:** Tomography, X-ray computed, Multiple trauma, Thoracic injuries, Mortality, Survival rate, Trauma centres

## Abstract

**Background:**

Thoracic injuries are a leading cause of death in polytrauma patients. Early diagnosis and treatment are of paramount importance. Whole-body computed tomography (WBCT) has largely replaced traditional imaging techniques such as conventional radiographs and focused computed tomography (CT) as diagnostic tools in severely injured patients. It is still unclear whether WBCT has led to higher rates of diagnosis of thoracic injuries and thus to a change in outcomes.

**Methods:**

In a retrospective study based on the trauma registry of the German Trauma Society (TraumaRegister DGU^®^), we analysed data from 16,545 patients who underwent treatment in 59 hospitals between 2002 and 2012 (ISS ≥ 9). The 3 years preceding and the 3 years following the introduction of WBCT as a standard imaging modality for the investigation of severely injured patients were assessed for every hospital. Accordingly, patients were assigned to either the pre-WBCT or the WBCT group. We compared the numbers of thoracic injuries and the outcomes of patients before and after the routine use of WBCT.

**Results:**

A total of 13,564 patients (pre-WBCT: *n* = 5005, WBCT: *n* = 8559) were included. Relevant thoracic injuries were detected in 47.8%. There were no major differences between the patient groups in injury severity (pre-WBCT: median ISS 21; WBCT: median ISS 22), injury patterns and demographics. After the introduction of WBCT, only minor changes were observed regarding the rates of most thoracic injuries. Clinically relevant injuries were pulmonary contusions (pre-WBCT: 18.5%; WBCT: 28.7%), injuries to the lung parenchyma (pre-WBCT: 12.6%; WBCT: 5.9%), multiple rib fractures (pre-WBCT: 10.6%; WBCT: 21.6%), and pneumothoraces (pre-WBCT: 17.3%; WBCT: 21.6%). The length of stay in the intensive care unit (pre-WBCT: 10.8 days; WBCT: 9.7 days) and in hospital (pre-WBCT: 26.2 days; WBCT: 23.3 days) decreased. There was no difference in overall mortality (pre-WBCT: 15.5%; WBCT: 15.6%).

**Conclusions:**

The routine use of WBCT in the trauma room setting has led to changes in patient management that are not reflected in the rates of diagnosis of severe thoracic injuries (e.g. tension pneumothoraces, cardiac injuries, arterial injuries). By contrast, there was a relevant increase in the rates of diagnosis of minor thoracic injuries, which, however, did not result in an improvement in survival prognosis.

## Background

Thoracic injuries account for 25% of deaths in polytrauma patients and are thus a common cause of death among these patients [1, 2]. In recent years, a trauma room algorithm has become widely accepted in Germany which is based on Advanced Trauma Life Support (ATLS^®^) and the European Trauma Course [3–5]. Nevertheless, the imaging technique to be used in the trauma room setting continues to be a matter of debate on account of time constraints, the level of radiation exposure, the patient’s overall condition, and other factors [6–8]. Computed tomography (CT), and in particular modern multi-slice computed tomography (MSCT), has become the standard imaging modality for diagnosing thoracic injuries [6–8].

After the usefulness of focused CT in addition to chest radiography had been demonstrated, the introduction of MSCT in 1998 led to a further substantial improvement in imaging techniques in the trauma room setting [9–13]. The technical advances associated with MSCT provided the basis for integrating whole-body computed tomography (WBCT), which is also referred to as a trauma scan, into the management of severely injured patients. In a recent review of the literature, Donaubauer et al. showed that the use of WBCT as a diagnostic tool had positive effects. They as well as other authors, however, did not specify the indications for WBCT [14, 15]. It is still unclear whether the replacement of traditional imaging (conventional radiography of the cervical spine, chest and pelvis and subsequent focused CT) by the trauma scan as the standard diagnostic imaging modality led to an improvement in the diagnosis of thoracic injuries. We conducted a retrospective analysis of the trauma registry of the German Trauma Society (TraumaRegister DGU^**®**^) in order to assess the number of diagnosed thoracic injuries before and after the introduction of WBCT as a standard imaging modality and to investigate whether the trauma scan led to a change in patient outcomes.

## Methods

The trauma registry of the German Trauma Society (TraumaRegister DGU^®^) provided the data used. The TraumaRegister DGU^®^ of the German Trauma Society (Deutsche Gesellschaft für Unfallchirurgie, DGU) was founded in 1993. The aim of this multi-centre database is the pseudonymised and standardised documentation of care for severely injured patients. Data are collected prospectively from the site of the accident until discharge from hospital. Included are patients who are admitted to hospital via the emergency room and subsequently receive intensive or intermediate care and patients who arrive at hospital with vital signs and die before admission to the intensive care unit. The infrastructure for documentation, data management, and data analysis is provided by the Academy for Trauma Surgery, which is affiliated to the German Trauma Society. Scientific data analysis is approved according to a peer review procedure established by the Committee on Emergency Medicine, Intensive Care and Trauma Management of the German Trauma Society. The participating hospitals are mainly located in Germany. Currently, approximately 25,000 cases from more than 600 hospitals are entered into the database per year. For hospitals associated with TraumaNetzwerk DGU®, the entry of at least a basic data set is obligatory for reasons of quality assurance [3, 16, 17].

### Patient groups and definitions

During the study period from 2002 to 2012, we analysed all cases of patients who were admitted to the trauma room with an Injury Severity Score (ISS) greater than or equal to 9. Patients who did not undergo immediate surgery or were not admitted to ICU were not included in this analysis of TR-DGU data.

For cases in which the box for ‘Whole-body CT’ in the TraumaRegister DGU^®^ data collection form was checked, we assumed that a whole-body trauma scan was performed as a primary diagnostic procedure. The TR-DGU defines WBCT as a combination of CT studies that produce images (or slices) of the body in a continuous manner and cover at least the region from the skull base to the pelvis. Neither the type of equipment used nor other details such as table feed per rotation are specified for this imaging procedure. In addition, data about the scanner type and the application of contrast agents are not provided.

The other diagnostic approach consists of traditional imaging that involves conventional radiography of the cervical spine, the chest and the pelvis, often followed by focused CT (e.g. cranial CT).

**Fig. 1:**
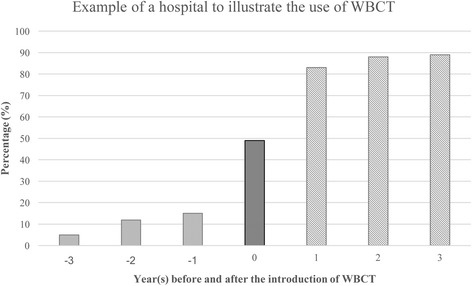
Example of a hospital to illustrate the use of WBCT on an annual basis. The bars show the percentage of patients whose data were entered into the TraumaRegister DGU^®^ and who underwent trauma scans (WBCT). Data for the years −3 to −1 were used to form the pre-WBCT group (traditional imaging) and data for the years 1 to 3 following the introduction of the trauma scan as a standard imaging modality were used to form the WBCT group. The year 0 is the year in which WBCT was introduced

The year in which the trauma scan replaced traditional imaging as the standard diagnostic imaging approach in the trauma room setting was determined individually for every hospital by two independent examiners *(HP* and *MK)*. When the two examiners disagreed, the opinion of a specialist in biometrics (*RL*) was obtained and was the final determination. The 3 years before (pre-WBCT group) and the 3 years after the introduction of the trauma scan (WBCT group) as a standard imaging procedure were analysed and compared (Fig. 1). The year in which the trauma scan was introduced was excluded from analysis since this year was usually a period of transition associated with a mixture of both imaging approaches. A maximum variation of 30% in both the pre-WBCT and the WBCT group provided the basis for decision. In addition, there had to be an increase in the MSCT rate by at least 50% or to at least 60% in the year following the introduction of WBCT when compared to the year preceding the introduction of the trauma scan.

### Inclusion and exclusion criteria

Only patients who underwent primary treatment at a regional (Level II) or supraregional trauma centre (Level I) (as defined by the TraumaRegister DGU^®^) were included [3, 16]. In addition, continuous documentation over a period of at least five consecutive years was required for inclusion. Hospitals were excluded when there were years for which no documentation was available or when the change in the number of cases was too drastic. A trauma scan rate of 50% or more in the first documented year was a further exclusion criterion since such a high rate suggested that this might not have been the year in which the trauma scan was introduced.

### Statistical analysis

Data that were obtained before and after the introduction of the trauma scan as a standard imaging modality for the assessment of trauma room patients were compared on the basis of percentages and means. Data were analysed using SPSS (version 22, IBM Corp., Armonk, NY, United States).

In consultation with a biostatistician affiliated to the TraumaRegister DGU^®^, we decided not to test between-group differences for statistical significance since even small differences that were of no clinical relevance were likely to be statistically significant on account of the large number of cases. For this reason, differences of medical importance were termed “relevant”. Trauma severity was assessed using the Injury Severity Score (ISS) and the New Injury Severity Score (NISS) [18]. Revised Injury Severity Classification (version II) (RISC II) scores were calculated in order to predict mortality, and thus to assess the probability of survival, at the time of hospital admission [19].

We presented the results in a descriptive manner in order to generate, but not test, a hypothesis. Continuous variables were presented as means (MV) and standard deviations (SD). Contingency tables were used to display frequency distributions presented as percentages. The 95% confidence intervals (95% CI) were calculated in order to assess the uncertainty of the means and percentages. In order to give a better insight into the distribution of the data, we calculated median value as well as the interquartile range in order to estimate the skewness, or symmetry, of the distribution where appropriate.

### Ethics and study registration

The present study is in line with the publication guidelines of the TraumaRegister DGU® and is registered as TR-DGU Project ID 2013–053 (http://www.traumaregister-dgu.de/fileadmin/user_upload/traumaregister-dgu.de/docs/Downloads/TR-DGU_-_Publikationsrichtlinie.pdf). As register data are assessed anonymously for scientific data analysis, individual informed consent is not required.

## Results

**Fig. 2:**
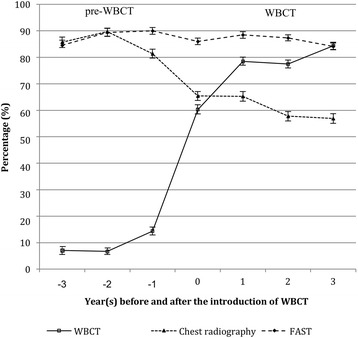
The figure shows a marked increase in the number of trauma scans after the introduction of WBCT. The change in the standard imaging approach is further demonstrated by the consecutive decrease in (conventional) chest radiography. A high proportion of patients underwent FAST. This proportion remained almost unchanged during the study period. The whiskers indicate the upper and lower limits of the 95% confidence interval

We were able to include 16,545 cases from 59 hospitals in our analysis. Of these cases, 5005 patients (30.3%) underwent traditional diagnostic imaging (pre-WBCT group) and 8559 patients (51.7%) had a trauma scan (WBCT group). The patients who underwent an imaging procedure in the year in which the trauma scan was introduced for routine use (*n* = 2981; 18.0%) were not included in this study because of a wide variety of implementation rates (Fig. 2). Table 1 provides an overview of demographic data, prehospital data, injury patterns, and injury severity.

**Table 1:** Demographic data, injury severities and injury patterns for the two patient groups. Mean and upper and lower limits of the 95% confidence intervals (CI) are given. Where appropiate, the median as well as the interquartile range (IQR) are given too

Parameter		Pre-WBCT group (*n* = 5002)	WBCT group (*n* = 8559)
Demographic data			
Male patients [%]	mean (95% CI)	73.5 [72.2–74.7]	73.0% [71.9–73.8]
Patient age [years]	mean (95% CI) median (IQR)	43.0 [42.5–43.6] 41 (25–59)	45.7 [45.2–46.1] 45 (26–62)
Patients with blunt trauma [%]	mean (95% CI)	94.8 [94.2–95.2]	94.5 [94.2–95.0]
Prehospital setting			
GCS	mean (95% CI) median (IQR)	11.0 [10.8–11.1] 14 (7–15)	11.1 [11.0–11.2] 14 (8–15)
Systolic blood pressure [mmHg]	mean (95% CI) median (IQR)	119.9 [119.0–120.9] 120 (100–140)	121.2 [120.5–122.0] 120 (100–140)
SpO_2_ [%]	mean (95% CI) median (IQR)	92.5 [92.2–92.9] 96 (92–98)	92.7 [92.4–93.0] 96 (92–98)
Patients with a GCS ≤ 8 [%]	mean (95% CI)	30.3 [29.0–31.6]	28.9 [28.0–29.6]
Patients with a systolic blood pressure ≤ 90 mmHg [%]	mean (95% CI)	18.2 [17.2–19.4]	17.9 [17.0–18.7]
Prognosis			
RISC II (predicted mortality) [%]		17.6	17.3
Patterns of injury			
Patients with an AIS_head_ ≥ 3 [%]	mean (95% CI)	48.9 [47.5–50.3]	48.7 [47.6–49.8]
Patients with an AIS_face_ ≥ 3 [%]	mean (95% CI)	2.4 [2.0–2.9]	4.2 [3.8–4.6]
Patients with an AIS_thorax_ ≥ 3 [%]	mean (95% CI)	44.1 [42.8–45.5]	50.0 [48.9–51.0]
Patients with an AIS_abdomen_ ≥ 3 [%]	mean (95% CI)	17.5 [16.4–18.5]	15.8 [15.0–16.6]
Patients with an AIS_extremities_ ≥ 3 [%]	mean (95% CI)	37.2 [35.9–38.6]	34.0 [33.0–35.0]
Patients with an AIS_soft tissues_ ≥ 3 [%]	mean (95% CI)	0.9 [0.6–1.1]	2.0 [1.7–2.3]
Patients without thoracic injuries [%]	mean (95% CI)	45.8 [44.4–47.2]	40.2 [39.2–41.3]
Injury severity			
ISS	mean (95% CI) median (IQR)	23.9 [23.5–24.2] 21 (14–29)	24.5 [24.2–24.7] 22 (14–29)
NISS	mean (95% CI) median (IQR)	29.4 [28.9–29.8]^*^ 25 (17–35)	30.2 [29.9–30.6]^*^ 27 (17–38)

*GCS* Glasgow Coma Scale, *AIS* Abbreviated Injury Scale, *(N)ISS* (New) Injury Severity Score, *RISC* Revised Injury Severity Classification

With the introduction of the trauma scan in the trauma room setting, the percentage of patients who underwent CT scanning increased from 73.7 to 92.8%. Once the trauma scan had become routine practice, 80.1% of all trauma room patients underwent whole-body MSCT – compared to 10.0% in the preceding years. At the same time, the percentage of patients who underwent conventional radiography of the chest decreased from 85.2 to 59.8% whereas the percentage of patients who had a Focused Assessment with Sonography in Trauma (FAST) examination remained almost unchanged (pre-WBCT group: 88.4%; WBCT group: 86.6%).

There were no major differences between the two patient groups in terms of injury severity, injury patterns and demographics. In both groups, the majority of patients were male (pre-WBCT group: 73.5%, 95% CI 72.2–74.7; WBCT group: 72.8%, 95% CI 71.9–73.8). The mean age of the patients in the pre-WBCT group (43.0 years, 95% CI 42.5–43.6) was slightly lower than that of the patients in the WBCT group (45.7 years, 95% CI 45.2–46.1). The mean ISS was 23.9 (95% CI 23.5–24.2) before and 24.5 (95% CI 24.2–24.7) after the introduction of the trauma scan as a standard imaging modality. The median ISS was 21 (IQR 14–29) versus 22 (IQR 14–29). The mean NISS was 29.4 (95% CI 28.9–29.8) in the pre-WBCT group and 30.2 (95% CI 29.9–30.6) in the WBCT group. The median NISS was 25 (IQR 17–35) versus 27 (IQR 17–38). In both patient groups, road traffic accidents were the main cause of injury (60.0% before and 58.2% after the introduction of WBCT). The vast majority of patients sustained a blunt thoracic trauma (94.8% in the pre-WBCT group and 94.5% in the WBCT group).

During the study period, the number of documented thoracic injuries increased (Table 2). Whereas the percentage of severely injured patients without thoracic injuries was higher in the pre-WBCT group (45.8%) than in the WBCT group (40.2%), the percentage of patients with relevant thoracic injuries (Abbreviated Injury Score - AIS_thorax_ ≥ 3) was lower before the introduction of the trauma scan as a routine imaging modality (44.1%) than after the routine use of WBCT (50.0%). Relevant changes were observed for the following thoracic injuries: injuries to the lung parenchyma (pre-WBCT: 12.6%; WBCT 5.9%), pulmonary contusions (pre-WBCT: 18.5%; WBCT: 28.7%), haemothoraces (pre-WBCT: 15.6%; WBCT: 14.0%), pneumothoraces (pre-WBCT: 17.3%; WBCT: 21.6%), multiple rib fractures and flail chest (pre-WBCT: 10.6%; WBCT: 21.6%), and injuries to the thoracic spine (AIS ≥ 2) (pre-WBCT: 10.9%; WBCT 13.2%).

**Table 2:** Thoracic injuries in the two patient groups

Parameter	Pre-WBCT group (*n* = 5002)	WBCT group (*n* = 8559)
Type of thoracic injuries		
Injury to the lung parenchyma [%]	12.6 [11.7–13.5]	5.9 [5.4–6.4]
Pulmonary contusion [%]	18,5 [17.4–19.6]	28,7 [27.7–29.7]]
Pneumothorax [%]	17.3 [16.3–18.4]	21.6 [20.7–22.5]
Tension pneumothorax [%]	2.9 [2.4–3.4]	2.5 [2.2–2.8]
Haemothorax [%]	15.6 [14.6–16.6]	14.0 [13.3–14.7]
Multiple rib fractures and flail chest [%]	10.6 [9.7–11.4]	21.6 [20.7–22.5]
Fractures of two ribs [%]	6.4 [5.7–7.1]	6.6 [6.1–7.1]
Fracture of one rib [%]	3.4 [2.9–3.9]	3.3 [2.9–3.7]
Arterial injury (thorax) [%]	1.5 [1.2–1.8]	1.5 [1.2–1.8]
Diaphragmatic injury [%]	1.0 [0.7–1.3]	1.1 [0.9–1.3]
Thoracic spine injury ≥ AIS 2 [%]	10.9 [10.0–11.8]	13.2 [12.5–13.9]
Thoracic spinal cord injury [%]	1.7 [1.3–2.1]	1.9 [1.6–2.2]
Cardiac injury [%]	0.4 [0.2–0.6]	0.5 [0.4–0.7]
Severity of thoracic injuries		
No thoracic injuries [%]	45.8 [44.4–47.2]	40.2 [39.2–41.3]
AIS_thorax_ = 1 [%]	2.8 [2.3–3.2]	2.0 [1.7–2.3]
AIS_thorax_ = 2 [%]	9.2 [8.4–10.0]	9.3 [8.7–9.9]
AIS_thorax_ = 3 [%]	24.9 [23.7–26.1]	28.2 [27.3–29.2]
AIS_thorax_ = 4 [%]	12.4 [11.5–13.3]	14.6 [13.8–15.3]
AIS_thorax_ = 5 [%]	4.7 [4.1–5.3]	5.3 [4.8–5.8]
AIS_thorax_ = 6 [%]	0.2 [0.1–0.3]	0.3 [0.2–0.4]

*AIS* Abbreviated Injury Scale

The mortality of patients who were managed before the routine use of the trauma scan was 15.5% (95% CI 14.5–16.5). Following the introduction of WBCT as a routine imaging modality, the mortality rate remained almost unchanged (15.6%, 95% CI 14.9–16.4). There was no relevant difference between the two groups in the probability of survival or mortality as predicted at the time of hospital admission. The RISC II score was 17.6% for the pre-WBCT group of patients and 17.3% for the WBCT group. The Standardised Mortality Ratio (SMR) was 0.88 before and 0.90 after the introduction of the trauma scan as a standard primary diagnostic imaging modality.

A relevant change in the percentage of patients with organ failure (pre-WBCT: 43.5%, 95% CI 42.0–45.0; WBCT: 43.9%, 95% CI 42.7–45.1) and multi-organ failure (pre-WBCT: 26.5%, 95% CI 25.2–27.8; WBCT: 26.9%, 95% CI 25.8–27.9) was not observed. The duration of ventilation (days of intubation), however, was longer before the routine use of the trauma scan than after the introduction of WBCT (pre-WBCT: 6.7, 95% CI 6.6–7.2; WBCT: 5.6, 95% CI 5.4–5.8). The same applies to the length of ICU stay (pre-WBCT: 10.8, 95% CI 10.5–11.2; WBCT: 9.7, 95% CI 9.4–10.0). The pre-WBCT group had fewer ventilator-free days during the first 30 days of hospital stay (19.8 days, 95% CI 19.4–20.1) than the WBCT group (20.8 days, 95% CI 20.6–21.0). The length of hospital stay decreased by approximately 3 days from 26 days (95% CI 25.5–26.9) in the pre-WBCT group to 23 days (95% CI 22.7–23.9) in the WBCT group. The percentage of patients who required a chest drain in the trauma room or operating room settings dropped from 20.0% before the routine use of WBCT to 18.5%. The length of stay in the trauma room decreased by 14 min from 78 min (95% CI 76.0–79.0) in the pre-WBCT group to 64 min (95% CI 63.0–65.0) in the WBCT group. Table 3 provides an overview of the results obtained as well as median and interquartile range where appropriate.

**Table 3:** In-hospital management and outcome parameters for the two patient groups. Mean and upper and lower limits of the 95% confidence intervals (CI) are given. Where appropiate, the median as well as the interquartile range (IQR) are given too

Parameter		Pre-WBCT group (*n* = 5002)	WBCT group (*n* = 8559)
Trauma room			
Intubation in the trauma room [%]	mean (95% CI)	55.6 [54.2–57.0]	49.3 [48.3–50.4]
Chest drain in the trauma room [%]	mean (95% CI)	20,0% [1, 9–21]	18,5% [3, 7–19]
Time spent in the trauma room [minutes]	mean (95% CI) median (IQR)	78 [76–79] 68 (51–87)	64 [63–65] 55 (40–77)
Chest radiography [%]	mean (95% CI)	85.2 [84.3–86.2]	59.8 [58.7–60.8]
Trauma scan [%]	mean (95% CI)	10.0 [9.2–10.8]	80.1 [79.3–81.0]
Time to trauma scan [minutes]	mean (95% CI) median (IQR)	31.3 [29.8–32.9] 29 (20–38)	23.6 [23.3–24.0] 21 (15–30)
Discontinuation of trauma room management because of emergency surgery [%]	mean (95% CI)	6.5 [5.8–7.2]	5.6 [5.0–6.1]
Surgery before ICU stay [%]	mean (95% CI)	44.0 [42.5–45.5]	40.5 [39.3–41.7]
Further management			
Length of ICU stay [days]	mean (95% CI) median (IQR)	10.8 [10.5–11.2] 8 (3–18)	9.7 [9.4–10.0] 6 (2–15)
Length of intubation/ventilation [days]	mean (95% CI) median (IQR)	6.9 [6.6–7.2] 3 (1–13)	5.6 [5.4–5.8] 2 (0–9)
Length of hospital stay [days]	mean (95% CI) median (IQR)	26.2 [25.8–26.9] 23 (11–37)	23.3 [22.7–23.8] 17 (9–30)
Ventilator-free days [days]	mean (95% CI) median (IQR)	19.8 [19.4–20.1] 24 (2–29)	20.8 [20.6–1.1] 27 (12–30)
Outcome			
24-h mortality [%]	mean (95% CI)	8.9 [8.1–9.7]	8.2 [7.6–8.8]
Hospital mortality [%]	mean (95% CI)	15.5 [14.5–16.5]	15.6 [14.9–16.4]
Organ failure [%]	mean (95% CI)	43.5 [42.0–45.0]	43.9 [42.7–45.1]
Pulmonary failure [%]	mean (95% CI)	26.2 [24.9–27.6]	22.2 [21.2–23.2]
Multi-organ failure [%]	mean (95% CI)	26.5 [25.2–27.8]	26.9 [25.8–27.9]

## Discussion

The objective of our study was to investigate whether the introduction of the trauma scan for the diagnostic evaluation of polytrauma patients in the trauma room led to a change in the rates of diagnosis of thoracic injuries. For this purpose, we compared the trauma scan approach and conventional radiography with or without focused CT for the diagnostic assessment of polytrauma patients. We placed particular emphasis on determining the year in which the trauma scan replaced conventional techniques as the standard diagnostic imaging approach individually for every hospital and formed the two groups of patients that were compared on the basis of these hospital-specific data. Our study showed that the routine use of the trauma scan did not lead to a change in the rates of diagnosis of severe thoracic injuries requiring immediate treatment (e.g. tension pneumothoraces, cardiac injuries, arterial injuries). By contrast, there was a relevant increase in the rates of diagnosis of minor thoracic injuries (e.g. pulmonary contusions, pneumothoraces, multiple rib fractures). The additional information provided by WBCT, however, did not lead to an improvement in survival prognosis. This and the following results regarding the diagnosed injuries must be interpreted carefully, because we could not rule out a change in the incidence of thoracic injuries during the study period.

Our findings are particularly noteworthy as the year 2002 has so far been used as the cut-off year for the introduction of WBCT in similar studies that were performed on the basis of the TR-DGU [20, 21]. In our study, however, the year in which the trauma scan was used as a standard imaging approach for the management of severely injured patients in the trauma room setting was determined individually for 59 hospitals and data for a total of 13,545 patients were analysed.

Previous studies showed that chest-focused CT detected clinically relevant injuries that had been missed with conventional imaging and that led to a change in their management. Both focused CT and conventional radiography are traditional imaging procedures that are used in the trauma room setting. Our study demonstrated limited superiority of the trauma scan over traditional imaging as a tool for diagnosing thoracic injuries in the trauma room. It is important to note that, unlike other studies that compared WBCT and chest radiography, our study compared a WBCT group with a pre-WBCT group that underwent both conventional chest radiography and organ-specific CT (chest CT). MSCT does not appear to provide a major diagnostic benefit when it comes to the diagnosis of severe acute life-threatening thoracic injuries such as cardiac injuries and tension pneumothorax. By contrast, the trauma scan appears to be superior to traditional imaging modalities in detecting minor thoracic injuries that are of less clinical relevance. Multiple rib fractures, pneumothoraces and pulmonary contusions can apparently be detected far more easily by a trauma scan. In our opinion, the decrease in the rate of diagnosis of injuries to the lung parenchyma can be explained by the fact that the majority of hospitals in which the trauma scan was introduced as a standard imaging modality in the trauma room have modern CT scanners that provide images of a better quality than older devices. Advanced equipment allows minor lung injuries to be identified more easily. For example, a small pneumothorax or a small pulmonary contusion can today be reliably detected and diagnosed. We believe that the large increase in the rate of diagnosis of multiple rib fractures is a result of the routine use of the trauma scan since undisplaced fractures are not obscured by overlapping anatomical structures and can be clearly visualised as well. The same applies to the increased rate of diagnosis of pneumothorax. Higher spatial resolution can explain both the decrease in diagnosed injuries to the lung parenchyma and the increase in diagnosed pulmonary contusions. There is, however, no direct evidence supporting the authors’ assumption that there were a high number of false diagnoses of injuries to the lung parenchyma in the group of patients who did not undergo WBCT.

The introduction of multi-slice CT and the associated marked decrease in scanning time resulted in a considerable reduction of breathing artefacts. In addition, technical advances led to an improvement in the overlap between adjacent slices and thus to a more accurate diagnosis. Another aspect to consider is that the modality on which diagnosis was based in the pre-WBCT group remained unknown in many cases. For example, the number of cases in which chest radiography, which has been found to be generally inferior to CT in detecting thoracic injuries, or chest-focused CT was used is unclear [9].

In 2002, Rieger et al. conducted a study comparing the role of the trauma scan with that of conventional chest radiography. Their results underline the superiority of the trauma scan. They reported that 18% of thoracic injuries were detected only by WBCT and that WBCT provided relevant information on the extent of injury in 78% of all lesions [22]. In 2004, Albrecht et al. examined 50 polytrauma patients using whole-body single-slice spiral CT. Forty-three of these 50 patients had additional chest radiographs. Whereas conventional chest radiography detected only 20% of all thoracic injuries (12 of 61 injuries), CT of the chest, which served as the gold standard, had a sensitivity of 100% in detecting thoracic injuries (75 of 75 injuries). Conventional chest radiography demonstrated only 2 of 13 pneumothoraces and 6 of 18 pulmonary contusions. By contrast, WBCT showed all 14 pneumothoraces and all 21 pulmonary contusions [23]. Although the study by Albrecht et al. was based on single-slice CT and not on multi-slice CT, it shows that CT is superior to chest radiography as an imaging modality and has a higher diagnostic accuracy for injuries that are likely to be of potentially less clinical relevance (e.g. a small pneumothorax or pulmonary contusion).

In 2007, Weninger et al. too investigated the diagnostic role of whole-body multi-slice CT. They retrospectively studied polytrauma patients (ISS ≥ 16) who had sustained at least one life-threatening injury to the head, chest or abdomen (AIS ≥ 4) and survived ICU admission. Two groups of patients were compared. One group underwent traditional imaging (conventional radiography, FAST and focused CT), the other had a trauma scan (whole-body multi-slice CT). In the trauma scan group, 92.9% of all thoracic injuries were detected. By contrast, only 38.3% of all thoracic injuries were diagnosed by conventional radiography, 13.6 by FAST and 76.5% by focused CT in the group of patients who underwent traditional imaging [24]. Like Rieger et al., however, Weninger et al. did not specify the types of thoracic injuries that the patients sustained. In our study, we used comparable inclusion criteria and obtained similar results.

A further aspect that should be considered is that ultrasound (FAST) continues to play an important role as a diagnostic imaging technique in the trauma room setting although the TR-DGU does not provide data on whether additional chest ultrasound provided information of high relevance. Nevertheless, FAST continues to be an important diagnostic modality that is used in addition to WBCT and has not been replaced by new imaging techniques.

A positive effect of the early use of WBCT on patient outcome and time management was proved for the first time by Weninger et al. [24]. and Hilbert in 2007 [25]. In 2009 and 2013, Huber-Wagner et al. analysed TR-DGU data and demonstrated a survival benefit for severely injured patients who underwent whole-body multi-slice CT [20, 21]. The results reported by Huber-Wagner et al. were confirmed by Kanz et al., who too reported in a study from 2010 that the early use of WBCT in polytrauma patients improved the probability of survival [26]. In a retrospective study from 2011, Wurmb et al. compared the use of whole-body multi-slice CT and traditional imaging (conventional radiography, FAST and organ-focused CT) as initial diagnostic tools in the management of polytrauma patients and the influence of these two approaches on outcome. Whereas the two imaging approaches were associated with similar mortality rates, patients who underwent a trauma scan had a higher mean ISS. This suggests a positive influence of the trauma scan on survival [27]. Hutter et al. and Kimura and Tanaka reported that the pattern of injury played a key role in decisions about imaging modalities. Both studies demonstrated a survival benefit for patients with blast injuries who underwent WBCT [28, 29].

In our study, we analysed TR-DGU data for 13,545 patients and found no reduction in mortality and a decrease in the length of ICU stay by only 1 day after the introduction of WBCT. Although the length of hospital stay was up to 3 days shorter with WBCT, we assume that this is not the result of a positive effect of changes in patient management in the trauma room setting since the pre-WBCT group showed an incidence of organ failure (43.5%) almost identical to that in the WBCT group (43.9%) (Table 3).

How can these different results that are based on the same data be explained? There were differences in the inclusion criteria that were used in our study and in the studies by Huber-Wagner et al. [20, 21]. Whereas Huber-Wagner et al. investigated only patients with blunt trauma and an ISS ≥ 16, we studied patients with blunt trauma (pre-WBCT group: 94.8%, WBCT group: 94.5%) and penetrating trauma and an ISS ≥ 9. An improvement in outcome can be more easily achieved in patients with more severe injuries. This is not only self-evident but was also confirmed in a follow-up study by Huber-Wagner et al. [21]. In our opinion, a further explanation for the different results may be the study period, which appears to play an important role. Whereas the studies by Huber-Wagner et al. are based on observation intervals from 2002 to 2004 and 2002 to 2009, our study used data from a considerably longer period, i.e. from 2002 to 2012. In our opinion, the period from 2002 to 2004 can be regarded as the early phase of WBCT diagnosis, i.e. a period during which a change in diagnostic imaging approaches took place. The introduction and implementation of WBCT in the trauma room setting was associated with modifications of trauma room algorithms. Apart from an optimisation of procedures, it appears possible that the introduction of a new imaging modality was not the only reason for an improvement in patient outcome but that other factors such as better training of trauma room personnel and more intensive care of patients played a role as well. Although WBCT is undoubtedly superior to traditional imaging modalities, modern trauma room procedures are of such a high quality (for example, as a result of the introduction of ATLS® training) that better imaging in the trauma room setting appears to have only limited potential for further improvement of outcome. A possible explanation for the increase in the probability of survival that was reported by Huber-Wagner et al. in their studies from 2009 and 2013 may be that those hospitals that introduced the trauma scan at an early stage (between 2002 and 2004) undertook intensive efforts to optimise the management of severely injured patients in the trauma room. In our study, we eliminated this effect by investigating every hospital individually. In 2015, Donaubauer et al. summarised the core statements of the S3 guideline that the German Trauma Society established for the treatment of patients with severe and multiple injuries and assessed the S3 guideline on the basis of a literature search. The authors found that many studies suggested a positive effect of whole-body computed tomography on the duration of care and survival. In addition, they as well as other authors reported an absence of clear indications for the use of WBCT [14, 15]. In our opinion, these findings reflect the results of our study in which we were able to show that traditional imaging in the trauma room, i.e. conventional radiography with or without additional focused CT, was not associated with a poorer outcome than the trauma scan (whole-body CT, usually whole-body multi-slice CT) in a non-selected patient population. It should be noted, however, that the trauma scan led to a relevant reduction in the time that was spent in the trauma room (i.e. from 78 min to 64 min). Sierink et al. conducted a prospective randomised multi-centre study and compared whole-body CT and conventional imaging modalities in terms of patient outcome and the time spent in the trauma room [30]. The study included 1083 trauma patients (mean ISS = 20) who were managed between 2011 and 2014. These patients underwent either immediate WBCT or conventional imaging and selective CT scanning. The authors found no significant difference in 30-day mortality (16% for both imaging approaches). The same applies to the results obtained in a subgroup analysis for patients with polytrauma and patients with traumatic brain injury. Similar to our study, Sierink et al. reported that the immediate use of WBCT led to a significant reduction in the time spent in the trauma room from 72 min to 63 min.

### Limitations

Our study is limited by its retrospective nature. In addition, it was difficult to identify a clear increase in the annual number of WBCT scans for some hospitals and thus to determine the year in which the trauma scan was introduced since the change in the diagnostic imaging approach is a complex process that does not take place on a single day. In addition, only the year but not the month in which a particular trauma case was managed must be entered into the TraumaRegister DGU^®^. For biometrical reasons, only complete years are therefore analysed.

Although efforts are undertaken by the TR-DGU to increase data completeness and data correctness as well as to include all severely injured patients (e.g. regular reports of data completeness, audits of all trauma centres regarding data correctness), up to 10% of all cases likely go unreported [31, 32]. A misclassification of the individual (thoracic) injury as well as the overall injury severity of the patients should be taken into consideration too [33].

Assuming that the trauma scan was introduced as a standard imaging modality when a modern CT scanner was bought and/or used instead of an older device, the increase in the rates of diagnosis of thoracic injuries may not only be associated with the imaging modality itself but may also be largely attributable to an improvement in scanning technology.

One reason for this unexpected result regarding the survival rate may be the inclusion criteria of ISS ≥ 9. Another reason could be the retrospective assignment of the year in which the trauma scan replaced traditional imaging as the standard diagnostic imaging approach in the trauma room setting.

A further limitation of the study is the absence of details regarding imaging protocols such as the type of scanner and the application of contrast agents, not to mention slice thickness, table feed per rotation, and the type of CT data reconstruction. These details were not taken into consideration in this study although they have a relevant effect on the quality of findings. Instead of asking hospitals for the year in which they introduced WBCT, we had to use biometric trends to determine the year in compliance with data protection laws. This is a further limitation of our study.

## Conclusion

Following the replacement of traditional imaging (conventional radiography and focused CT) by WBCT as the standard imaging modality in the trauma room setting, a higher number of thoracic injuries were detected. The majority of these cases, however, were minor injuries requiring no immediate treatment. There was no change in the clinical management of the thoracic injuries investigated here. During the period from 2002 to 2012, the routine use of the trauma scan did not improve survival in the non-selected patient population (ISS ≥ 9). WBCT, however, led to a relevant reduction in the time spent in the trauma room (i.e. from 78 to 64 min).
